# Clinical application of liquid biopsies to detect somatic *BRCA1/2* mutations and guide potential therapeutic intervention for patients with metastatic breast cancer

**DOI:** 10.18632/oncotarget.27863

**Published:** 2021-01-19

**Authors:** Neelima Vidula, Leif W. Ellisen, Aditya Bardia

**Affiliations:** ^1^Massachusetts General Hospital, Harvard Medical School, Boston, MA, USA

**Keywords:** cell-free DNA, *BRCA1/2*, PARP inhibitor, metastatic breast cancer, plasma based genotyping

## Abstract

Plasma based genotyping via cell-free DNA may identify actionable mutations for potential therapeutic intervention in patients with advanced malignancies including breast cancer. In this article, we discuss recent studies using cell-free DNA testing to identify and classify somatic *BRCA1/2* mutations in metastatic breast cancer, and potential future applications for the treatment of metastatic breast cancer.

## INTRODUCTION

In recent years, plasma based genotyping via cell-free DNA (cfDNA) or “liquid biopsy” has emerged as a robust means to detect actionable mutations and guide genotype-directed therapies for patients with advanced malignancies [1]. For metastatic breast cancer, the utility of cfDNA in identifying actionable mutations has been demonstrated [2], and recent studies have validated the feasibility of tumor genotyping for targeted treatment matching [3]. Notably, a PI3K inhibitor has now been approved for *PI3KCA* mutant hormone receptor positive (HR+)/HER2- metastatic breast cancer using diagnostic cfDNA testing to identify *PI3KCA* mutations [4]. CfDNA offers the advantage of being less invasive and possibly more sensitive than tumor tissue genotyping assays [5, 6].

PARP inhibitors have recently been approved as a targeted therapy for the treatment of germline *BRCA1/2* mutant metastatic breast cancer based on results from two pivotal phase III clinical trials. The phase III OlympiAD [7] and EMBRACA [8] studies demonstrated significant improvement in progression-free survival with olaparib and talazoparib, respectively, compared to chemotherapy, for metastatic breast cancer. PARP inhibitors may also improve patient reported outcomes and quality of life. However, germline *BRCA1/2* mutations only account for about 5–10% of breast cancer [9], limiting their broad applicability.

A question that arises is whether PARP inhibitors may also be beneficial in metastatic breast cancer with somatic *BRCA1/2* mutations. In a recent study led by our team [10], we demonstrated the emergence of somatic *BRCA1/2* mutations detectable by cfDNA, largely in the absence of germline *BRCA1/2* mutations. In our analysis of 215 patients undergoing cfDNA testing as part of care for metastatic breast cancer, we observed that 29 (13.5%) had somatic *BRCA1/2* mutations detectable in cfDNA, which were seen in various subtypes, and often clonal in nature. Four percent had somatic *BRCA1/2* mutations that were known germline-pathogenic, and the rest were novel variants, based on classification using extrapolation from reputable genomic databases. In addition, we demonstrated increased sensitivity to a PARP inhibitor in a CTC culture derived from a patient with a pathogenic somatic *BRCA1* mutation, highlighting the unique role of PARP inhibition for patients with somatic *BRCA1/2* mutant breast cancer. However, not all somatic *BRCA1/2* mutations are functionally significant, i.e., pathogenic. For example, in a CTC culture derived from a patient with a novel variant *BRCA2* mutation, we did not observe any impact of a PARP inhibitor. Interestingly, this patient also had widespread expression of the APOBEC mutation signature that encompassed the *BRCA2* mutation itself, suggesting the novel *BRCA2* mutation was likely a passenger mutation rather than a driver mutation. Based on these findings, we developed an algorithm to guide clinical assessment of somatic *BRCA1/2* mutations, as well as designed a genotype-directed clinical trial for patients with metastatic breast cancer [11].

A genotype-directed clinical trial is currently ongoing at our institution and other academic centers to determine the efficacy of PARP inhibition in somatic cfDNA *BRCA1/2*-mutant metastatic breast cancer [11], and the results of this trial may help expand the population of patients who benefit from PARP inhibitors, similar to what has been observed in ovarian cancer. A combined analysis of two studies evaluating a PARP inhibitor in ovarian cancer demonstrated similar efficacy in germline *BRCA1/2* mutant patients and somatic *BRCA1/2* mutant patients [12].

Besides detection of germline and somatic *BRCA1/2* mutations, cfDNA analysis also allows for detection of reversion *BRCA1/2* mutations [13]. The acquisition of *BRCA1/2* reversion mutations is a well described phenomenon [14], which restores the open reading frame (and function) of the *BRCA1/2* gene, thus rendering a PARP inhibitor ineffective. In a second multicenter analysis [13], we demonstrated that routine plasma-based genotyping can be utilized to classify *BRCA1/2* cfDNA mutations as germline, somatic or reversion mutations, based on specific loci and the mutant allele fraction of the *BRCA1/2* mutation. Of 828 patients with advanced malignancies including breast cancer undergoing testing with a 73 gene cfDNA assay, one or more pathogenic *BRCA1/2* mutation was identified in 7.2% of patients, and both somatic and germline variants were detected. Polyclonal reversion mutations were found in 21.4% of patients with germline *BRCA1/2* mutations, most often in association with receipt of a prior PARP inhibitor.

Another study found that the absence of pre-existing cfDNA *BRCA1/2* reversion mutations in patients with ovarian cancer who had somatic or germline *BRCA1/2* mutations and were treated with rucaparib was associated with improved progression-free survival [15]. Therefore, the identification of cfDNA *BRCA1/2* reversion mutations may have important implications for therapeutic response to PARP inhibitors, and will be studied in our ongoing clinical trial of a PARP inhibitor for somatic *BRCA1/2* mutant metastatic breast cancer [11]. A complementary ongoing trial is also evaluating the efficacy of the PARP inhibitor, olaparib, in somatic *BRCA1/2* mutant metastatic breast cancer, with initial results suggesting potential efficacy [16].

In summary, plasma-based genotyping is a promising strategy for the detection of *BRCA1/2* mutations and could potentially guide triage to genotype-directed matched therapy with a PARP inhibitor. Ongoing studies will help determine the therapeutic utility of this approach and impact on long term clinical outcomes for patients with metastatic breast cancer.
